# Medical Benevolent Funds

**Published:** 1909-02-20

**Authors:** 


The Hospital

A Journal of

The Medical Sciences and Hospital Administration.

New Series. No. 103, Vol. IV. [No. 1175, Vol. XLV.] SATURDAY, FEBRUARY 20, 1909.

### Medical Benevolent Funds.

If medical men are not as long-lived as the clergy and the lawyers, their disadvantage is at any rate not extreme. But what does, from the point of view of provision against ill-health or accident, differentiate so greatly the position of the medical practitioner from that of his fellow professional men is the essentially personal nature of his practice and his work. The stockbroker, the solicitor, the accountant, and others in like occupations, as well as the tradesman, can afford to take a few days or weeks off work in case of sickness or overstrain without necessarily suffering any material pecuniary loss. The office and the managing clerks and the general routine of business still remain at work; and the absence of the principal for a short time, while it may not be good for business, is at least not immediately followed by its complete cessation. The medical man's capital lies in his professional knowledge and ability and in his personality. His skill and learning cannot be separated from him, and without physical health and energy his capital soon disappears. Acquired at great expense during the very long and arduous training now necessary to equip a doctor with the ever-increasing armament of scientific knowledge against disease, this capital is of so precarious and personal a nature that any one of a thousand circumstances over which no foresight or prudence can establish any control may in a moment disperse it to the winds.

How, then, may members of the medical profession best face this awkward but undeniable fact regarding their pecuniary prospects in life? By the same means that anyone else adopts to avert the consequences of possible and unavoidable disaster—that is, by insurance. We cannot estimate every factor that is going to tell for or against us in our struggle for existence: some we do not know of, some are not valid for our consciousness, as Dr. Claye Shaw put it in a lecture on the psychology of success which was reported in these pages last year (The Hospital, August 22, 1908, p. 543). But we can at least do something to avert utter and unmerited destitution for ourselves or our dependents in years to come should calamity strike at the roots of prosperity. These remarks are suggested by the recent annual reports of certain medical benevolent funds which partake of the nature partly of insurance and partly of charity. The British Medical Benevolent Fund distributes £2,450 to 124 annuitants, none of whom is under sixty years of age, and last year an almost equal sum was expended in grants to those for whom no annuity could be provided. Even then the treasurer's report states that many deserving cases were passed over for want of funds for their relief. With the object of bringing up all the annuities to the standard of £26 per year a special appeal was made for further endowment funds to the extent of £20,000. The Bishop of Oxford attended the meeting at which the treasurer's statement was read, and addressed it in a speech full of the sympathy and insight into the peculiar difficulties and hardships of a doctor's life which would be expected of his father's son. Dr. Paget declared that there is no profession in which a man is compelled, without any choice of his own and without any chance of escape, to run greater risk of impoverishment for himself and his family than in the medical profession. The Lord Mayor was equally emphatic in maintaining that the State and the public have done very little in return for the benefits which the medical profession has conferred upon the country. We trust this admirable charity will benefit largely by the publicity which the recent meeting obtained for its needs and its merits.

Then, again, there is the Society for the Relief of Widows and Orphans of Medical Men, which last month voted over £1,200 as the half-yearly relief of a number of widows and orphans of medical men. This excellent society partakes less of the nature of a charity than does the Benevolent Fund, and in that sense it has perhaps an even stronger appeal to us than the latter. Relief is granted only to the widows and orphans of deceased members. When we state that the invested funds of the Society approach six figures, we make it clear how stable is its position and how well worthy of support from all members of the profession who have wives or children. With regard to other recent reports and meetings of certain mutual and other assurance societies which especially do business with medical men, we need say nothing except that, one and all, they emphasise the point we have already pressed home—that is, the precarious nature of the doctor's prosperity, depending as it does on the maintenance of health and strength in an occupation which often makes exacting demands upon both. To support the funds we have alluded to is both a praiseworthy work of charity and also a practical and sensible deed of self-protection. Further, we think there is much to be said in favour of Sir Douglas Powell's suggestion that many medical men have opportunities, of which they should take advantage, of recommending these philanthropic societies to the notice of the public. After all, the profession fights the battle against disease and death; the State or the public might at least provide ambulances for those who are wounded in the fray.

